# Audience Interpretation of Risks in Health Promotion Campaigns About Underage Drinking: Qualitative Interviews With Parents of Adolescents

**DOI:** 10.1002/hpja.70184

**Published:** 2026-04-16

**Authors:** Hoang Van Nguyen

**Affiliations:** ^1^ Centre for Alcohol Policy Research (CAPR) La Trobe University Bundoora Australia

**Keywords:** adolescent, alcohol, health promotion, parents, underage drinking

## Abstract

**Issue Addressed:**

Underage alcohol use has been linked to risks of physical, mental, and social harms to young people. Despite the known risks, research shows that parents may choose to supply alcohol to their children on occasions for various reasons. This has prompted several health promotional campaigns aimed at parents to discourage the practice of parental supply of alcohol, but there has been little evidence of how the messages are received by the target audiences, i.e., parents of adolescents.

**Methods:**

Grounded in the existing literature on alcohol‐related harms, social dimensions and communication of risks, the current paper conducted a qualitative analysis of interviews with parents of adolescents to understand their interpretation of risks in a series of Australian health promotion campaigns that addressed underage drinking.

**Result:**

The study demonstrated how target audiences brought in their own lived experiences and social worldviews to interpret and internalise messaging about risks in ways that are nuanced and situational.

**Conclusions:**

The findings demonstrated how parents' lived experiences and worldviews influenced their interpretation and alignment with the health promotion messages about parental supply of alcohol. While the ways the parents negotiated with the health promotion messages may not be scientifically‐grounded, it was not always due to unawareness of risks but based upon strategies and assessment of risks in situational contexts.

**So What?:**

Understanding of how lived experiences inform interpretation of health promotion campaigns has implications for more effective alcohol‐related risk communication aimed at behaviour change to reduce alcohol‐related harms among young people.

## 1 Introduction

### 1.1 Underage Drinking, Risk and Parenting

Underage alcohol use has been linked to major adverse outcomes and is advised against [1]. Underage drinking has decreased in the past decades, which has been shown to be attributable in parts to parents' behaviours, including increased monitoring and control in supplying alcohol to adolescents [2, 3, 4]. The emphasis on parents' responsibilities in managing underage drinking is in line with the theoretical view of risk management being a dominant part of contemporary parenting [5]. Risk discourses surrounding children has paradoxically become adult‐centred, as the parents' lived experiences and worldviews (rather than the child's own) become central to the ways child‐related risks are perceived and managed [6].

While parents are the default risk managers, the child is not always a passive ‘recipient’ of adult protection and control, but at times, a competent social actor [5], who has been shown to form understanding of risk and risk management [7, 8, 9]. This is particularly true for older adolescents aged 15 and above, who are often characterised as ‘risky’ to themselves or others as they take on risk‐taking behaviours and pushing the boundaries with alcohol use [10]. Moreover, parents can sometimes become a source of risk to children, at times unknowingly due to being unaware of the risks [11]. Particularly, parents sometimes choose to supply alcohol to their adolescent children [12, 13, 14]. Parents may allow or supply alcohol for adolescents to consume under adult supervision, with the assumption that alcohol use under supervision reduces potential risks [12, 13, 15]. Parents may also supply alcohol to their children if they know or assume that other parents do so [16]. However, some studies suggested that parents may overestimate how accepting the broader community is of underage drinking and how common parental provision of alcohol is [14], which influences their own attitudes and behaviours [13].

### 1.2 Risk Discourses in Health Promotion About Underage Drinking

As alcohol use, particularly of adolescents, is often discussed in terms of health risks and risk management strategies [1, 17], the current study focuses on risk discourses in health promotion campaigns that address this issue. Risk discourses are defined as the shared knowledge of risk that is constructed in and through the use of language and other meaning‐making resources in social interactions [18]. Risk discourses influence the ways we understand and manage risks [19], often in the form of mass communication for public health issues [20].

Public health advertising in the mass media has been extensively used to reach a large part of the population, often focusing on changing personal behaviours (e.g., drinking, etc.) among others [21, 22]. In the form of advertising, health risks are mass communicated by experts and/or governments to educate the uninformed public [23], a framing which hegemonizes expert knowledge to identify and manage risks in society [18]. Public health advertising is often underpinned by the assumption that awareness of risk leads to avoidance of risky behaviours, thus compelling the public to self‐govern and self‐regulate their behaviours accordingly—most frequently observed in health risks associated with ‘lifestyle choices’, such as alcohol use [23, 24]. The target audiences are more likely to act on public health messages and change their personal behaviours when they perceive the messages to be relevant and translatable into personal actions [24]. Health promotional campaigns are also more likely to succeed in influencing behaviour changes when they are concurrent with other factors to support key messages in the campaign, such as creation and enforcement of policies, availability of and access to key services and products [22, 25].

### 1.3 A Previous Textual Analytical Study on Risk Discourses in Health Promotion

There have been a number of studies looking at risk discourses in health communication and media, which provided insights on how risk information is communicated and influences people's health‐related behaviours and actions [26]. One such study was a previous work by the current author [27], who investigated the construction of risk discourses in a series of public health advertisements named ‘*Alcohol. Think Again*’, which targeted parents of adolescents to raise their awareness of alcohol harm and reduce alcohol supply to young people. The study was a textual analysis of three advertisements (referred to as Advertisement A, B and C), which formed a major part of and represented the campaigns (see Table 1). The textual analyses investigated the use of language, images and sounds to form a cohesive message about the risks of underage drinking to encourage the ‘correct’ behaviours in parents and the community, i.e., to say ‘no’ to supplying alcohol to adolescents [27]. The findings demonstrated how the advertisements used language, images and sounds to construct various discourses of risk to warn the public of the risks of underage drinking, and invoke a sense of individual responsibility of parents and collective responsibility of the community to control said risks (see Table 1 for summary of the advertisements).

**TABLE 1:** Risk discourses present in ‘Alcohol. Think Again’ advertising campaigns.

Campaign	Summary of content	Main discourses of risk [27]
Campaign A—‘I see’	Featuring a son asking his father for alcohol and the father considering it, followed by scenes showing potential harms for young people, including road accidents, injuries, psychological harms, and brain damage, which are reported by people in professional roles (medic, doctor, psychologist and taxi driver)	Use of dark and grim depictions of risks to create a ‘semiotics of fear’ that ‘scares’ parents into doing the right thing [28], alongside scientific risk discourses which highlighted the role of expertise in identifying and managing risks [18, 19]
Campaign B—‘I need you to say no’	Featuring scenes of young people directly asking the audience to be parent and say ‘no’ to providing alcohol to under 18 s—even when they ask for alcohol, bargain with the parents, and act out when being denied	Use of ‘governmentality’ perspective which highlighted self‐governance and self‐responsibility [18] with an adult‐centred view [6] to emphasise each individual parent's responsibilities in controlling risks, while also highlighting the ‘risky’ and risk‐taking nature of adolescence which require adult control [10]
Campaign C—‘We all need to say no’	Featuring a daughter asking her father for ‘some’ alcohol and the father saying ‘no’ at a family dinner, followed by a metaphorical scene where other adults appear in the background behind the family and start praising the father for saying ‘no’	Framing alcohol supply control in terms of the cultural unacceptability of risk behaviours [29] to highlight saying ‘no’ to alcohol supply as the 'acceptable' risk behaviour in this local culture

However, the intended message in health promotional materials may or may not be embraced by the target audiences [30]. Previous research had pointed out that some parents, who allowed their children a little alcohol under supervision, considered themselves to be different from the parental figures in underage drinking advertisements who supply alcohol to their children, and thus, considered themselves not part of the target audiences of such campaigns [12]. As the target audiences' interpretations of risk in health promotion may differ from what was intended, there is a need to further investigate how health promotional materials about underage drinking are interpreted by their audiences [27]. As such, to bridge this gap, the current paper aims to examine the risk discourses in public health advertising about underage drinking as interpreted by its target audience. While findings from the previous textual analyses [27] are referenced, the present study is distinct in its aims and methodological framework, focusing on how the messages about risk are interpreted by the audiences, rather than discursively constructed in the health promotional materials.

### 1.4 Audience Interpretations of Advertising

Understanding how the audience interpret media texts has been discussed at lengths in media studies [31]. One major theory was the Encoding/Decoding framework to analyse the intended messages ‘encoded’ in the media and how its audiences ‘decode’ these messages [32]. While there is a dominant preferred meaning encoded in the media text's structures, readers can decode it in three positions based on their lived experiences. The positions include: (1) the ‘dominant position’ where they agree with the preferred meaning as it reflects their experiences, (2) the ‘oppositional position’ where they reject the preferred meaning which does not represent their experiences, and (3) the ‘negotiated position’ where they generally accept the dominant interpretation, but resist and modify it to represent their experiences [32, 33]. The Encoding/Decoding framework, thus, recognises the active roles of the audience in interpreting and consuming media texts [31]. Using the Encoding/Decoding framework, Morley [33] looked at how audiences' interpretations of media texts varied by basic socio‐demographic factors (i.e., age, sex, race and class), and by identification with the education system and subcultures.

Similarly, the social semiotic rhetorical approach to communication theorised the roles of the ‘rhetor’ and ‘interpreter’ in making meanings [34]. The rhetor produces the text with a ‘preferred’ meaning, while interpreters interpret the text by directing their attention to certain aspects and details of the text to understand and internalise its meanings and potentially form further communicative actions. The text only becomes meaningful when the interpreter directs their attention to and engages with aspects of the texts as such [34]. It is the audience's ‘interest’, i.e., the condensation of their social experiences in awareness of the social environment and backgrounds at the moment of interpretation, that directs their engagement with the texts and shapes their positionality vis‐à‐vis the texts. Instead of investigating varying interpretations of different socio‐economic groups as per Morley's study [33], the social semiotic framework accounts for past experiences that may shape and influence the interpretation of texts [34, 35].

One method to study interpretation of media texts is through interviews with the audience. Through in‐depth interviews, participants demonstrate how they realistically consume media, as they bring in their understanding of the local contexts, lived experiences and worldviews to make sense of media texts [36, 37]. In other words, interviews with audiences provide insights into the process of interpretation, rather than making assumptions about what the audiences truly think [36, 38]. A number of studies have used interviews to investigate audience interpretation of risk discourses in media texts in specific contexts; for example, one study investigated how Vietnamese women brought in their lived experiences to interpret public health advertisements about child helmet in Vietnam [39]. Interviews, thus, add a layer of understanding of the sociocultural contexts and experiences that influence the target audience's understanding.

## 2 Methods

To examine audience interpretation of risk discourses in public health advertising about underage drinking, the current paper presents a case study of audience interpretation of the ‘*Alcohol. Think Again*’ campaigns. Building upon the findings from the textual analyses of the same advertisements in the previous study [27], the current paper focuses on interpretation by parents of adolescents in Australia, who are the target audiences of the advertisements, by conducting semi‐structured interviews with parents of at least one adolescent child who lived in Australia. Ethics approval for the project was granted through the Human Research Ethics Committee at La Trobe University (HEC23213).

Participants were recruited using a paid advertisement via La Trobe University's social media, through which potential participants expressed their interest in participating in the research and provided their contact details via an online form. Further information about the research was provided via email to those who expressed interest. Interviews were then organised with 10 participants. Most participants were female, and living in Melbourne, Victoria (see Table 2). The over‐representation of participants living in Melbourne could potentially be due to the nature of social media advertising, which might have been more visible to those living in the same city as the La Trobe University whose social media was used to recruit.

**TABLE 2:** Demographic characteristics of participants.

Demographic characteristics	*N* = 10
Gender	
Man	1
Woman	9
State in Australia	
Victoria	8
New South Wales	1
Northern Territory	1
Remoteness	
Capital city	7
Regional city	3
Number of adolescent children	
1 adolescent child	7
2 adolescent children	2
3 adolescent children	1
Cultural backgrounds	
Culturally and Linguistically Diverse (CALD)	3

The interviews were conducted via video call, which allowed the interviewer to remotely show the video advertisements to participants. Participants provided their informed consent, which was audio‐recorded, prior to the interviews. The interviews were audio‐recorded and later transcribed for analysis. The transcripts were de‐identified, and pseudonyms were given to each participant (used throughout this paper).

During the interviews, the participants were asked to watch three video advertisements A, B and C (see Table 1), and then answer questions about their interpretations of the advertisements, as well as the particular language, images and sounds that they noticed and attended to as a basis for their interpretation, as guided by the social semiotic rhetorical approach [34]. They were then asked questions about their interpretations of the advertisements in relation to their own beliefs, values and lived experiences about underage drinking as a parent. The focus of this paper was not to evaluate how well‐received, effective or impactful the advertisements were (see [40]), but to explore the ways in which the audiences brought their lived experiences to decode the intended messages of the advertisements. Nonetheless, the advertisements' effectiveness was mentioned to prompt discussions about positionality (i.e., whether the participants agreed with, opposed to, or negotiated with the intended public health messaging), and how their lived experiences may have influenced their perception of the advertisements.

The interview data was then coded in NVivo 15 [41] to identify the dominant, oppositional and negotiated positions using the Encoding/Decoding framework [32], to understand how participants decoded the dominant meanings encoded in the advertisements. Rather than analysing the data by socio‐demographic characteristics of participants, the analysis adopted a social semiotic perspective [34] to consider the meaning‐making resources participants drew their attention to in the moment of watching the advertisements, and how their lived experiences, personal values and beliefs may have influenced their interpretations. From there, a thematic analysis was conducted to identify emerging themes in each position.

## 3 Findings

The section presents the emergent themes followed by the audiences' positionality based on the Encoding/Decoding framework [32]. Of note, no evidence of oppositional position was found, as all participants were generally in agreement with the public health messages (i.e., dominant position) or in partial agreement (negotiated position).

### 3.1 Theme 1: Risks of Underage Drinking

#### 3.1.1 Dominant Position: Recognition of Risks of Underage Drinking

All participants said their attention was drawn to the portrayals of risks of underage alcohol use in the advertisements. For example, all participants stated they immediately noticed the physical harm as a result of intoxication, such as road injuries due to drunk driving, or damage to the brain in Advertisement A. Some participants described feeling emotional after watching the advertisements due to the imagery of road accidents and injuries.

I was surprisingly distressed by it. I got goosebumps and I thought I was going to cry… I don't think I was ‘triggered’ by it, but I can relate to it. I thought it was a powerful ad to describe and illustrate the effects that alcohol can have on young people. (Allison)

Some participants also pointed out the use of imagery related to medical and health problems, such as the CT scanner or scans of the human brain, which were potential risks of underage drinking that they did not immediately think of. The use of medical‐related imagery was generally well regarded by most of the participants, as they placed trust in the advice from medical professionals as portrayed in Advertisement A. The high regard of medical professionals, thus, suggested conformity to the scientific discourse of risk, which prioritise medical expertise as the source of health risk knowledge [18, 19].

All the parents in the study stated they were aware of the potential risks of underage drinking, and in agreement that adolescents must not engage in heavy and risky drinking. They also related the health promotional messages to their real‐life experience as parents, stating that they also did not allow or supply alcohol to their children, with a few exceptions (the negotiation of which will be explored in a later section).

#### 3.1.2 Negotiated Position: Nuances in Portrayals of Risks

Some parents hold negotiated positions with the portrayals of alcohol‐related risks in the advertisements, where they acknowledged the potential harm of underage drinking, but resisted how it was portrayed.

Firstly, despite acknowledging the harm of underage drinking, some participants resisted the use of grim imagery of injuries and medical settings to portray the severe risks related to underage drinking [27]. The dark, grim visual portrayals of risks to constitute a ‘semiotics of fear’ which were used to ‘scare’ parents into doing the ‘right’ thing, that is, not providing alcohol to their children [25, 28]. Shock factor has been shown to significantly increase attention and positively influence behaviour of audiences [42]. In this case, the ‘shocking’ imagery did attract all participants' attention; however, some participants in the current study considered it to be too ‘graphic’ or triggering, which they said they would rather avoid than engage with.

Parents' past experiences of underage alcohol use may also influence their perception of risk [6]. Particularly, some participants raised their concern that to people who engaged in underage drinking but did not experience the severe risks when they were younger, the portrayals may potentially seem like ‘a stretch’ or ‘scare tactic’, which they may dismiss.

While the advertisements portrayed alcohol‐related risks in any quantity [27], concern was raised about how people who had strong assumption about alcohol harm being related to the quantity of alcohol consumed may misinterpret or not pay attention to such details. Particularly, a few parents pointed out that some people, especially adolescents, may associate injuries from road accidents, fights, or vomiting, with excessive drinking exclusively. They noted that some teenagers may assume that they could take the risk and consume alcohol, but not experience the harm portrayed in the advertisement, if they did not let themselves get to the point of being heavily intoxicated.

They'll get to a point where they'll drink but they won't vomit, [like] I won't get myself in a position where I'll have an ambulance picking me up or end up in hospital. (Amanda)

Similarly, some parents also noted many people could associate the risks of brain damage (portrayed by medical imaging such as X‐Ray or CT scans in Advertisement A) as the consequences of excessive drinking over longer periods of time. Such assumption may lead to some teenagers or their parents thinking that the risks portrayed in the advertisements do not apply to their children if they only drink a little alcohol occasionally.

I still don't think it sends the message, the message that how harmful alcohol is to the brain, because unless they understand that it is happening to them whatever amount of alcohol they're drinking—if that's not happening to them, they'll be thinking they're not impacted. (Amanda)

### 3.2 Theme 2: Parental Responsibility to Control and Monitor Underage Drinking

#### 3.2.1 Dominant Position: Acknowledgement of Parent Responsibility

All participants were in agreement with the advertisements and acknowledged that it is parents' responsibility to prevent and monitor their children's alcohol use, in line with the literature on risk prevention as parents' responsibility [5].

… we [parents], until the kids are old enough, are making a lot of their decisions on their behalf. (Kimberly)

Most parents also stated that they related to the struggles of parents to prevent underage drinking. For example, the adolescents in Advertisement B were portrayed as rebellious, arguing with their parents and pushing boundaries (e.g., bargaining, saying their parents also drank when they were their age, slamming the door and yelling). Some participants said they related Advertisement B for addressing the struggle of parents to say ‘no’ as they may risk damaging parent–child relationships, which had been shown to be a motivator for parental alcohol supply [43, 44]. The parents found the advertisements relatable to their own experiences which increased their alignment with the dominant intended messages and perceived effectiveness [24], especially when the advertisements emphasised the negotiation and decision‐making that happened when parents needed to make the ‘right’ decisions to prevent risks for their children.

[The advertisement] is effective, and it addresses the angle that I love, because I can feel that struggle, and it's a real struggle for parents when we deal with teenagers, because it's really a challenge to say no. (Mai)

One parent, Kimberly, also noted that she felt that the portrayals of adolescents being individuals with autonomy and awareness of the risks of underage drinking and asking parents for help in Advertisement B sent a powerful message. It emphasised how, despite pushing boundaries at times, adolescents wanted parents to reinforce said boundaries and protect them from harm, which Kimberly and some other participants resonated with.

#### 3.2.2 Negotiated Position: Older Adolescents' Agency and Accountability

Whilst all generally agreed that parents had the responsibility to monitor and control supply of alcohol to their teenaged children, some participants interpreted that the advertisements put the accountability or blame on parents for their adolescent children's drinking, which they partially resisted. These participants put an emphasis on the adolescents' agency, as they may choose to consume alcohol regardless of their parents' efforts to prevent underage drinking, thus holding them more accountable for their own actions:

I don't particularly like the blaming, putting the responsibility on the parent exclusively. I don't appreciate that because we're not talking 10‐year‐olds, 12‐year‐olds, we're talking maybe 15, 16, 17‐year‐olds, at least from what they look like in the ad, who could have a bit more responsibility or could be held more accountable. […] It seems to be a little bit leaning towards just one side and not acknowledging that maybe teenagers should also be held more accountable on their own decisions once they leave the house. (Gabriela)

In other words, some parents in the study acknowledged their teenaged children's agency to have their own risk knowledge and management strategies [7], more so than what was portrayed in the advertisements. This view also demonstrated the diminishing parental responsibilities for older adolescents around misuse of substances [10].

### 3.3 Theme 3: Social Acceptability and Cultural Norms Related to Underage Drinking

#### 3.3.1 Dominant Position: Refusal to Supply Alcohol to Adolescents as a Social Norm

Some parents' attention were reportedly drawn to the tagline ‘2 out of 3 parents are already saying no’, which sent a message about saying ‘no’ being a social norm that they aligned with. Some also noted and appreciated the portrayals of multiple adults saying ‘no’ and praising the father in Advertisement C who said ‘no’ to his child who asked to try alcohol at the dinner table. One of these parents said the portrayals of saying ‘no’ as the norm gave them reassurance and confidence that they were doing the ‘right’ thing and that other parents were also not allowing alcohol. The advertisement, thus, discredited the perceived appropriateness of the act [45].

Some parents noted that the statistics gave them confidence, as they had assumed parental alcohol supply was more commonplace. Under this assumption, some parents said they may struggle to say ‘no’ because they did not want to be the only parent in their social circles doing so. The experiences they shared support the previous literature that parents find it harder to say ‘no’ to supplying alcohol to their children if they know or assume other parents are doing so [13, 16].

The use of statistics in a public health advertisement series constructed a view of the social norms in Australia surrounding underage drinking and parental alcohol supply. A few parents also noted, by pointing out that many parents in Australia are not providing alcohol to their children, the health promotional message could be helpful for parents who are new migrants in Australia and unfamiliar with the culture. For example, Jessica, who worked with migrant parents, noted that these parents may appreciate the information about the social acceptability around underage drinking in Australia, which they may not be familiar with:

[New migrant parents] might be a little bit confused or uncertain as to what the kids are being exposed to, or what other families think are acceptable in Australia. […] I think that sends a really good message that, no, most people don't let their kids do that. (Jessica)

Similarly, Gabriela, who self‐identified as a migrant, noted that since the drinking culture in Australia is different from that of her home country, she had learned and adjusted her parenting style to be more in line with the cultural expectations regarding underage drinking in Australia.

For us, [underage drinking] is more frowned upon here than it is in [our home country]. I think we were a bit [stricter] with our kids living here than if we had been in [our home country]. (Gabriela)

Thus, the participants took into consideration the varying drinking cultures, that is, the norms around drinking practices and patterns that are enforced within societal groups [46], both of themselves and others when interpreting the advertisements. The emphasis on the drinking ‘norms’ in Australia was said to be one way that health promotion campaigns could help new migrant parents and subsequently, their teenaged children, to understand the norms of an unfamiliar culture where they are socialising into.

#### 3.3.2 Negotiated Position: Social (Un)acceptability Surrounding Underage Drinking

However, some parents partially resisted the use of statistics and portrayals of community approvals and social acceptability in the advertisements as a means of persuading parents. For some, the issue was with the statistics, stating that while ‘2 out of 3’ was not an overwhelming majority and may not be convincing enough:

If you told me 90% of parents did that then I'd feel a little bit naughty being in the 10%. But if—to tell me two thirds of parents don't let their kids drink and one third does—it's not really a strong message to me. (Melissa)

A few participants also pointed out that in the multicultural society of Australia, families may come from various cultural backgrounds with different drinking cultures, where it is common for adolescents to first try alcohol with their families [47]. Drawing from their experiences and observations of other families in their social circles, these participants noted that people from these cultural backgrounds may resist the hardline stance of saying ‘no’ to adolescents having their first drink with their family, as the common practice in their cultures may be linked with positive emotions and family‐oriented values [47]. For example, Gabriela noted that in her culture and family, underage drinking in supervised settings was more socially acceptable than portrayed in the advertisements. Given her cultural experiences, she was less aligned with the message of the advertisements and was more open to allowing teenagers a ‘taste’ in supervised environments with their parents, thus demonstrating how cultural and family backgrounds had an influence on the ways the parents interpreted the advertisements. Nonetheless, rather than taking an oppositional stance against the advertisements, she negotiated with the dominant message, stating that she adjusted her parenting to fit with the dominant drinking culture in Australia (see quote above), which demonstrated a negotiation of different drinking cultures in how she interpreted the public health messages as well as in her daily life. Interestingly, not all participants drew from their cultural backgrounds to interpret the advertisements, as two other participants who identified as having CALD backgrounds did not bring in their cultural experiences with drinking as part of the discussions. In other words, demographic characteristics were not the determining factor across the audiences' interpretations, but rather, their individual experiences of cultures that influenced their understanding of the advertisements.

### 3.4 Theme 4: Applicability of Health Promotional Messages About Parental Alcohol Supply

#### 3.4.1 Negotiated Position: Flexibility in Situational Contexts

Some parents in this study said while they understood the risks of underage drinking and generally agreed with the public health messaging of not providing alcohol to their children, they negotiated with the applicability of the main message in some situations. Even though they understood and aligned with the health advice about no alcohol should be supplied to people under 18, the parents said they may struggle when their children grow closer to the legal drinking age, have more autonomy and may choose to engage in risky behaviours regardless of parental control [10, 16].

As such, some parents in the interview said if they could not stop the teenaged children from taking risks and used alcohol anyway, they might have to consider going against the health advice and allowing it in a supervised environment to mitigate the risks [13, 15, 16, 44], despite knowing of the harm of alcohol to adolescents. The reluctant acceptance was grounded in a perceived realism of the (presumed) risky nature of adolescence [44]:

I think I know rationally that no alcohol is better. That alcohol damages the teenage brain and it's not actually safe. [But] it's safer than them sitting on a beach or in the car and getting smashed to the point of unconsciousness, all the vulnerabilities that go with that. I know it's not safe, but I think it's a last resort, [if] you can't hold a line about ‘no’, then it's kind of like a minimisation measure. (Rebecca)

Rather than being uninformed of the harm of underage drinking, the decision to allow drinking under adult supervision was based upon thoughtful consideration of all potential risks, both long‐term (health issues and brain damage) and short‐term (immediate vulnerabilities and dangers). Even when the decision contradicted scientific and evidence‐based advice (i.e., no alcohol to under 18 s), it is not entirely lacking in rationality and reasoning, but an ad‐hoc strategy to manage risks and uncertainties in the situation [48].

In addition, a few parents noted to them, allowing their teenaged children to drink under supervision was not only mitigating the risks, but also beneficial to the adolescents, as the parents could teach them about moderate consumption [13]. To these parents, supplying alcohol in a supervised environment was considered part of ‘alcohol socialisation’, a process by which young people familiarises themselves with alcohol and learns about the values connected to moderate and low‐risk alcohol use [47]. The benefits of drinking with family also brought into the discussion the notion of pleasure and enjoyment associated with drinking practices [17, 49], rather than solely focusing on the negative consequences:

At least in my family, it worked better to know that [alcohol] is there, it's going to be around you, and it's better if you know how to drink and how to enjoy drinking in moderation and know when and up to what point. (Gabriela)

Some parents also remarked that the scenario in Advertisement C, where the young woman was asking her father to “have some” of what he was drinking, was more realistic and likely to happen in their lives than that of Advertisement A, where the young man was asking his father for permission to “grab a few beers” with his friends. Thus, these parents felt more related to Advertisement C and placed themselves in the role of the parent in this scenario, as they might have to deliberate if their child was asking to ‘have a sip’, making a clear distinction between that and ‘drinking alcohol’ [12, 16]. To them, letting adolescents have a taste or try some alcohol in a supervised environment was considered more ‘acceptable’ than letting them have a glass of wine or a bottle of beer. For example, Allison said some parents in her social circle may allow their children to have a taste under supervision:

… the people that I've known [with children] close to my daughter['s age], I would say half of them were very clear on no drinking, and half of them were quite chill about it, as long as it was at home with a parent, and it was like tasting—not like, ‘oh, let's have a beer together’. No, you can try the beer at 15, 16; you cannot have a whole beer by yourself. (Allison)

As such, most parents displayed strong awareness of the risk of underage drinking, but also said they assessed the risks in each situation [12]. The risk assessment took into consideration specific circumstances and nuances in various situations, including the children's age and risk behaviours, the situations and environments where drinking may happen, the amount of alcohol, to decide whether to allow their children alcohol to some extent. These considerations are also not fixed, but rather context‐dependent and evolving as their children age. Nonetheless, the messages about not supplying any amount of alcohol to adolescents were generally understood by participants, who took them into account to assess their real‐world situations.

So, [my son] has not had any alcohol, he's 14. I know that there might be a time before he's 18 and he's young and he's at home and might ask for something and I might give him a sip, but that's what this ad is telling me not to do. So now I'm thinking about it. (Allison)

## 4 Discussion

Overall, all the parents in the study acknowledged the alcohol‐related risks portrayed in the advertisements and agreed with the overarching message of not providing alcohol to their teenaged children in the advertisements. The advertisements created an ‘ideal’ selfhood and community, where everyone is a responsible parent and member of the community by not supplying alcohol to underage children [27]. How the audiences interpreted and aligned with the messages was evidently shaped by their own lived experiences and worldviews, which not only influenced what their attention was drawn towards, but also how they supported, challenged or negotiated with this ‘ideal’ selfhood and community in the health promotional messages [34, 50]. The participants not only drew from their own lived experiences, but also their observations of other people with different experiences, as evident in discussions about how the intended messages could be understood by people with different experiences, understanding and beliefs about underage drinking. The interviewed parents generally aligned with the intended messages of the advertisements, particularly about the harm of underage drinking, parents' responsibility and social norms to refuse supplying alcohol to adolescents. While no participant held an oppositional position, some partially resisted, modified and negotiated with the messages to reflect their own views, experiences, values and beliefs as well as their observations and knowledge of others', which led to nuances in the ways they perceived the risk discourses in the public health messages.

Firstly, participants drew from their experiences and observations to note that some people may assume the portrayed risks in the advertisements are linked to only frequent, long‐term excessive drinking, which may lead to further assumption that the advertisements were not aimed at them, as long as they did not engage in or allow heavy drinking [12]. In other words, prior assumed knowledge about alcohol‐related harm may influence the audience's perception of the risks being portrayed. While the current advertisements attempted to portray alcohol as harmful in any quantity [27], some participants pointed out that those with assumed prior knowledge may not take note of the nuanced details to interpret the message as such.

Secondly, while most agreed that it was the parents' responsibilities to prevent underage drinking, considering risk management as a central part of parenting [5, 6], some negotiations in the portrayal of responsibility and accountability were present in the interviews. Some parents considered adolescents to have agency and risk knowledge to partially take responsibility for their own actions, including engaging in risk taking behaviours such as alcohol use [51], especially outside of the homes and beyond parents' control. This view was more prevalent among the parents of older adolescent children in the study, whose parenting experiences potentially influenced how they acknowledged the agency and accountability of older adolescents when partially resisting the intended message.

Depicting saying ‘no’ to parental alcohol supply as the social norms was generally accepted by most participants as a meaningful construct to persuade themselves and other parents to do the same, which support previous research about social norms being a factor influencing an individual's behavioural change [25]. The health promotional messages based upon social approvals gave them assurance and confidence that saying ‘no’ is the ‘right’ thing to do, while discrediting assumptions about how common underage drinking and parental alcohol supply are in Australia [45]. However, social acceptability may not always be meaningful to the audiences, when the statistics were not overwhelmingly powerful. In addition, what is considered to be socially acceptable may differ in different drinking cultures with different social norms surrounding alcohol [46]. The differences in culture may lead to some resisting the health messages, whilst some may appreciate the insights into the cultures where they are socialising into.

Finally, while all parents generally agreed that parents should not allow their children to drink, there were negotiations regarding circumstances where they might make an exception. This is particularly true for parents of older adolescents, whose risky behaviours were considered inevitable [10, 44]. Allowing their teenaged children to drink in a supervised environment was considered a last resort to mitigate risks, but also a way to teach them about drinking in moderation [13]. For others, the amount of alcohol was a deciding factor, as having a taste of an alcoholic drink could be acceptable under some circumstances [16]. While the advertising campaigns had addressed that alcohol may cause harm in any quantity [27], there remained negotiation from the parents about this message, as most expressed that they had to consider various factors and circumstances in the specific scenarios, despite being aware of the risks. Thus, the parents can be said to be ‘reflexive’ to alcohol‐related risks, i.e., being self‐aware, actively and continually examining and interrogating risks in their surroundings and actions [52]. The parents' risk responses may not always be grounded in scientific or expert advice and thus, may appear ‘irrational’; however, they were not simply due to being misinformed or lacking awareness, but based on ‘reasonable’ strategies with careful considerations, to manage risks and uncertainties in their situational contexts [48].

As such, the meanings of public health advertisements were actively interpreted and negotiated through the individual's positioning of themselves in relation to the discourses of risk and alcohol use, thus highlighting the agency and intentionality in risk perception and behaviours [53]. Their consideration included not only alcohol‐related risks and risk management strategies, which are the usual focus of public health advertisements, but also more personal dimensions, such as parent–child relations, drinking cultures, social acceptability, alcohol socialisation, pleasure associated with moderate drinking, as they had discussed in the interviews.

## 5 Conclusion

The interviews with participants who were part of the intended target audiences, thus, provided insights into how the advertisements were interpreted and internalised in relation to one's lived experiences and beliefs, which has practical implications for risk communication in health promotion. How the risks of underage drinking were communicated in the public health advertisements proved to align with most participants' experiences as parents; however, some nuances could be considered in future iterations, such that the audiences will not disregard the message because they feel it was not aimed at themselves, but at ‘others’ who consume more alcohol and over longer periods of time than themselves or their children. Interviews with the participants also pointed out that sociocultural factors (such as drinking cultures and social acceptability), or more positive dimensions of drinking (pleasure, enjoyment and socialisation) were aspects to be taken into consideration to convey the health promotional messages more effectively, as these proved to have some impact on how the intended messages may be interpreted and negotiated with. In short, given the influence of lived experiences and personal beliefs in audiences' interpretations of risk meanings, communication of risk information should consider how laypeople's responses to risks may not always be based on scientific and expert advice, but can be guided by cultural beliefs, emotion‐driven preferences, and lived experiences [54, 55] or reasonable strategies based upon situational rationality [48]. As such, risk communication, especially of public health issues, should rely not only on scientific evidence, but also understanding of real‐world social experiences to convey fact‐based information more effectively [56].

It is also worth noting the limitations of the study. The limited number of participants means the findings are limited in generalisability, especially as the majority of participants were female and living in Victoria, due to recruitment bias via social media. Thus, the findings did not fully capture the range of experiences of people of all genders across Australia. Furthermore, the participants were recruited by expressing interest to an advertisement; thus, there might have been a recruitment bias towards those who had a strong opinion towards underage drinking, so findings may not represent the general public's views.

Nonetheless, the paper has explored the nuances of audience interpretations, while capturing emerging themes that provided understandings of parents' views of underage drinking in public health advertising. More research into audience interpretation, particularly with other groups of audiences (e.g., adolescents), will contribute to better understanding of alcohol‐related risks in health promotion, to improve their impact and influence behaviour changes to reduce alcohol‐related harm among young people.

## Funding

This work was supported by the Australian Rechabite Foundation (ARF).

## Ethics Statement

Ethics approval for the project was granted through the Human Research Ethics Committee at La Trobe University (HEC23213).

## Conflicts of Interest

The author declares no conflicts of interest.

## Data Availability

The data that support the findings of this study are available on request from the corresponding author. The data are not publicly available due to privacy or ethical restrictions.
